# Classification of Factors Affecting Manipulation Tendencies Using Decision Trees

**DOI:** 10.3390/bs16060998

**Published:** 2026-06-15

**Authors:** Seçil Ömür Sünbül, Müzeyyen Soyer

**Affiliations:** Department of Educational Sciences, Faculty of Education, Mersin University, Mersin 33343, Türkiye; secilomur@gmail.com

**Keywords:** manipulation tendency, self-esteem, attachment styles, decision tree

## Abstract

This study aimed to examine variables associated with manipulation tendency levels in adults and to describe current patterns using a decision tree method as a classification-based analytical approach. The research sample consisted of 543 adults (358 women, 65.93%; 185 men, 34.07%) residing in Turkey, aged 18 to 45 years (M = 25.79, SD = 4.23). Data were collected using a researcher-developed personal information form, the Manipulation Scale in Human Relations, the Rosenberg Self-Esteem Scale, and the Relationship Scales Questionnaire. The total composite score of the Manipulation Scale was used as the outcome variable and is referred to throughout as “manipulation tendency.” Manipulation tendency scores were dichotomized into low versus high groups using a median split to facilitate C&RT-based classification. Classification and Regression Tree was used to examine the hierarchical structure of variables related to manipulation tendency levels and to identify classification patterns among study variables. The decision tree approach was used not merely as an alternative statistical technique, but as an interpretable classification framework capable of identifying hierarchical and conditional pathways associated with manipulation tendency. Data were stratified-randomly split into training and test sets (70/30), and tree complexity was tuned via cross-validation using cost-complexity pruning. Model performance indicated acceptable classification accuracy within this sample, with a test-set accuracy of 0.81 (sensitivity = 0.74, specificity = 0.88, precision = 0.86, F1 = 0.79) and training accuracy of 0.86. The findings indicated several influential variables in classifying manipulation tendency levels, ranked by importance: preoccupied attachment style, self-esteem, age, dismissive attachment style, gender, secure attachment style, and fearful attachment style. Preoccupied attachment style was identified as the most salient variable in differentiating between high and low manipulation tendency groups. The decision tree structure showed that younger adults with higher preoccupied attachment scores were more frequently classified into the high manipulation tendency group. Self-esteem emerged as the second most influential variable, with lower self-esteem levels being more commonly observed among individuals classified in the high manipulation tendency group. Age also played a notable role in classification, with higher manipulation tendency classifications occurring more frequently among younger individuals. Dismissive attachment style contributed to the differentiation of manipulation tendency levels, particularly within specific attachment and age profiles. Gender-related patterns indicated that men were more frequently classified into higher manipulation tendency groups, especially among individuals with low self-esteem. Overall, the findings highlight the multifactorial and hierarchical nature of manipulation tendency classifications. They contribute to the literature by showing how attachment-related characteristics, developmental factors, and psychological variables jointly differentiate manipulation tendency profiles. These findings highlight the value of decision tree modelling for translating conventional psychological predictors into interpretable classification profiles of manipulation tendency.

## 1. Introduction

The ability to manage and influence others’ emotions represents a critical dimension of interpersonal functioning that can manifest in both adaptive and maladaptive forms (Austin & O’Donnell, 2013). As a specific form of emotional influence, manipulation involves the strategic use of social and cognitive abilities such as persuasion and strategic thinking to deliberately change, influence, and exploit the interpersonal environment (Jones & Paulhus, 2011). This phenomenon is defined as an intentional and often covert form of social influence that aims to alter others’ perceptions or behaviors through deceptive or coercive psychological strategies (Braiker, 2004). In this process, the manipulator aims to undermine the victim’s conscious and consensual decision-making by weakening the victim’s will, creating cognitive distortions, applying emotional pressure, or manipulating perceptions (Bursten, 1972). Furthermore, manipulators tend to act in line with their own interests and desires, using the other person’s vulnerabilities, emotional weaknesses, or lack of knowledge to push them toward a desired direction (Buss et al., 1987; Jones & Paulhus, 2011). Therefore, a core characteristic of manipulation is that it involves deception, emotional pressure, or an intention to control, typically to the detriment of the victim (Braiker, 2004). Within this broader framework, manipulation tendency refers to an individual’s overall propensity to engage in interpersonal manipulation across a range of conceptually related tactics, including the strategic use of emotions, deception, and other persuasive strategies to influence others’ judgments and behaviors.

Among these tactics, one of the most extensively studied and clinically salient forms involves strategies that target the individual’s emotions and emotional vulnerabilities (Braiker, 2004). These manipulative tactics typically originate through gradual and insidious processes of escalation (Bursten, 1972), characterized by the manipulator’s use of covert strategies that make initial detection particularly challenging for victims (Braiker, 2004). Specifically, the manipulator attempts to direct the other person according to their own desires by exploiting the victim’s emotional vulnerabilities, fears, feelings of guilt, love, empathic capacity, or value system (Clempner, 2017). Common tactics include gaslighting, love bombing, guilt-tripping, playing the victim role, emotional blackmail, silent treatment, humiliation, and criticism (Braiker, 2004). The primary function of these manipulative strategies is to create a power imbalance in the relationship and to enable the manipulator to gain control through systematic erosion of autonomy (Stark, 2007). Individuals characterized by manipulative tendencies are generally self-centered, narcissistic, or have low self-esteem, while also exhibiting characteristics such as lack of empathy and difficulty in setting boundaries (Bereczkei, 2018). In the present study, manipulation tendency is considered as an individual-level interpersonal tendency rather than clinically defined coercive control, and is operationalized as a composite construct that integrates emotional manipulation with four other interrelated tactics (aggressive manipulation, victim selection, self-concealment, and strategy use) assessed by the Manipulation Scale in Human Relations (Yılmaz, 2018).

To explain how manipulation tendency arises and persists in interpersonal contexts, it is important to consider both individual characteristics and relational tendencies. In line with this view, the present study uses a classification model that includes attachment styles as relational schemas, alongside self-esteem and key demographic variables (age and gender). This approach aims to clarify the hierarchical and potentially interacting contributions of these variables to manipulation tendencies.

Among these variables, attachment styles, which reflect how individuals perceive themselves and others in close relationships, are addressed as one of the key variables whose relationship with manipulation tendency is examined in this research. Attachment style refers to relatively stable psychological patterns that guide individuals’ expectations, emotions, and behavioral strategies in interpersonal contexts (Ein-Dor et al., 2011). Developed through early caregiving experiences, these patterns influence intimacy, trust, and emotion regulation across romantic, social, and professional relationships in adulthood (Mikulincer et al., 2005; Fraley & Roisman, 2019). In this respect, attachment styles provide an important theoretical framework for understanding how individuals manifest manipulation tendencies in interpersonal relationships.

The most critical developmental period in the formation of attachment style is considered to be infancy and early childhood (Mikulincer & Shaver, 2016; Fraley & Roisman, 2019). Attachment theories emphasize that interactions established with caregivers during this period form the basis of cognitive-emotional representations that individuals develop about themselves and others, and that these representations transform into relatively stable attachment tendencies over time (Mikulincer & Shaver, 2016; Fraley & Roisman, 2019). While the caregiver’s sensitive, consistent, and accessible responses to the child’s needs support the development of a sense of security and effective emotion regulation strategies, insensitive, inconsistent, or rejecting caregiving experiences increase the likelihood of insecure attachment patterns such as mistrust, avoidance of intimacy, or excessive dependency (Mikulincer et al., 2003). In the literature on adult attachment, attachment styles are mostly conceptualized based on the individual’s self-perception and expectations toward others, and are defined through different combinations of these two dimensions (Mikulincer & Shaver, 2007, 2016). Within this framework, secure attachment is characterized by relatively positive representations of both the self and others, reflecting confidence in one’s own worth and in others’ availability and responsiveness. Securely attached individuals tend to approach close relationships with trust and reciprocity and demonstrate effective emotion regulation in interpersonal contexts (Shaver et al., 2019). In contrast, insecure attachment patterns are linked to dysfunctional interpersonal strategies such as avoidance of intimacy, excessive approval seeking, or conflicting tendencies in relationships. Dismissive attachment tendency is associated with the individual developing positive expectations toward themselves but negative expectations toward others, with a tendency to maintain emotional distance and overemphasize independence in close relationships (Mikulincer & Shaver, 2016). Preoccupied attachment tendency is characterized by the individual having negative representations about themselves but positive representations about others, with intense proximity, approval, and reassurance seeking (Mikulincer & Shaver, 2016). In fearful attachment tendency, individuals hold negative expectations about both themselves and others, which leads to conflicting relational behaviors characterized by a simultaneous desire for intimacy and avoidance of closeness. This internal conflict is associated with interpersonal instability and increased vulnerability to personality pathology, making relational functioning particularly challenging for individuals with insecure attachment patterns (Meyer et al., 2001).

In this context, another fundamental variable addressed in the research is self-esteem, which refers to individuals’ general perceptions of self-worth, acceptance, and competence (Orth & Robins, 2014). Self-esteem is conceptualized as a relatively stable self-evaluation reflecting how individuals appraise their own value, competencies, and importance, and it is widely regarded as a key component of psychological well-being (Orth & Robins, 2022). Contemporary theoretical and empirical research indicates that self-esteem is not merely a subjective emotional state (Orth & Robins, 2022), but rather a psychological resource associated with individuals’ capacity to cope with stress (Orth et al., 2012), functioning in interpersonal relationships (Murray et al., 2006), and emotional resilience (Orth et al., 2018).

Building on this framework, the present study focuses on how cognitive structures such as attachment styles and self-esteem—both of which are theorized to develop early in life—are associated with manipulation tendencies in adulthood. Attachment styles are conceptualized as cognitive–emotional schemas that play a central role in shaping interpersonal expectations and behaviors. Using a decision tree classification approach, this study examines the hierarchical contribution of attachment styles and self-esteem to the differentiation of manipulation tendency levels. In doing so, the findings provide evidence regarding how variations in attachment-related characteristics and self-esteem are jointly associated with manipulation tendency profiles, thereby contributing to the literature without implying causal effects.

When the literature is examined, numerous studies have been conducted to understand the psychological characteristics and consequences experienced by individuals exposed to manipulation and coercive control (Stark, 2007; Sweet, 2019). Research indicates that victims of manipulative and psychologically abusive relationships frequently experience diminished autonomy, erosion of identity, and heightened emotional distress (M. A. Dutton et al., 1999; Stark, 2007). Akiş and Öztürk (2021), who emphasize that individuals exposed to manipulation often display low self-confidence and low self-esteem, argue that these vulnerabilities may increase the likelihood of being targeted as victims. They further state that such individuals are prone to feelings of helplessness, worthlessness, and guilt, and are more susceptible to threats and deception. Over time, continued exposure to manipulation may lead to a weakening of the individual’s sense of self and identity. Similarly, Tekiner (2022) emphasizes that manipulative individuals tend to select victims who appear psychologically vulnerable or powerless. Furthermore, Bursten (1972) notes that individuals characterized by intense feelings of inadequacy, excessive approval-seeking, difficulty asserting boundaries, and avoidant coping strategies may be more susceptible to manipulative dynamics. Overall, the literature suggests that exposure to manipulation and psychological control is associated with reductions in self-esteem, impaired self-confidence, increased depressive and anxiety symptoms, feelings of inadequacy and loss of control, and difficulties in social and relational functioning (M. A. Dutton et al., 1999; Sweet, 2019).

On the other hand, research directly addressing the self-esteem and attachment style profiles of individuals exhibiting manipulative tendencies is scarce. When the relevant literature is examined, studies on manipulators are mostly addressed with variables such as the dark triad (Şenyuva & Yavuz, 2024), cognitive distortions (Esin, 2022), childhood traumas (Tekiner, 2022), narcissism (Bursten, 1972), socio-emotional control (Nagler et al., 2014), empathy (Grieve & Panebianco, 2013), relationship satisfaction and self-compassion (Kayıtmaz, 2024), self-sabotage (Köse, 2019), belonging (Çınar et al., 2022), and differentiation of self (Şen, 2023). Although some research has investigated the associations of attachment styles and self-esteem with broader personality traits and suggested their potential relevance to manipulative tendencies (Bylsma et al., 1997), empirical studies systematically examining how manipulative tendencies vary across attachment styles and levels of self-esteem remain extremely limited (Schotman, 2022). Accordingly, the present study aims to contribute to the literature by identifying distinct psychological profiles associated with manipulative tendencies based on self-esteem and attachment-related variables, rather than by proposing causal explanations.

Despite the growing body of research on manipulation tendency, notable gaps remain regarding the joint and hierarchical examination of psychological and demographic variables associated with manipulation tendencies. Previous studies have generally focused on isolated associations, offering limited insight into how attachment styles, self-esteem, and basic demographic characteristics collectively differentiate individuals with varying levels of manipulation tendency. This limitation is important because manipulation tendency may not be explained by a single psychological variable, but rather by combinations of relational insecurity, self-evaluation, and demographic characteristics. From this perspective, attachment styles may shape individuals’ expectations, emotion regulation strategies, and responses to interpersonal threat, whereas self-esteem may influence how individuals evaluate themselves and respond to perceived rejection, inadequacy, or relational vulnerability (Mikulincer & Shaver, 2016; Orth & Robins, 2022).

The novelty of the present study lies in three connected contributions rather than in the introduction of new psychological predictors. Methodologically, the C&RT framework identifies higher-order interactions directly from the data and yields a single, rule-based classification hierarchy—a combination that regression with interaction terms (which requires a priori specification and assumes linearity), latent profile analysis (which produces probabilistic classes rather than explicit decision rules), and random forest models (which sacrifice a single traceable pathway) do not jointly provide in an interpretable form. Substantively, the model reframes manipulation tendency as a configurally organized tendency rather than the additive consequence of separate predictors, showing that preoccupied attachment, self-esteem, and age operate along hierarchically ordered pathways and that gender contributes only within a narrow conditional cell rather than as a uniform between-group difference. In applied terms, the resulting tree offers a preliminary, hypothesis-generating screening and classification framework, expressed as explicit if–then decision rules that can be evaluated and refined in independent samples.

To achieve this, a C&RT model was used to classify adults’ manipulation tendency levels based on secure, fearful, preoccupied, and dismissive attachment styles, self-esteem, age, and gender. The analysis aimed to determine the most influential predictors, the hierarchical order in which they operate, and the specific combinations that increase the likelihood of belonging to low or high manipulation tendency groups, thereby offering a clear, data-driven depiction of underlying psychological and demographic patterns.

In line with this aim, the study was guided by four research questions. First, which attachment-related, self-evaluative, and demographic variables contribute most to the classification of low and high manipulation tendency levels? Second, how are attachment styles, self-esteem, age, and gender hierarchically organized in the C&RT model when classifying manipulation tendency levels? Third, which combinations of attachment styles, self-esteem, age, and gender are associated with low and high manipulation tendency classifications? Fourth, what classification performance does the C&RT model demonstrate in distinguishing between the low and high manipulation tendency groups?

### C&RT Decision Tree Analysis as a Classification Approach

C&RT decision tree analysis was selected as the analytical framework because the main purpose of this study was not only to determine whether attachment styles, self-esteem, age, and gender were associated with manipulation tendency, but also to identify how these variables jointly classify individuals into low and high manipulation tendency groups. C&RT is a non-parametric tree-based method that recursively partitions the data into increasingly homogeneous subgroups according to the outcome variable, thereby producing a hierarchical classification structure (Breiman et al., 1984; Lemon et al., 2003). This feature is consistent with the present study because manipulation tendency may emerge through combinations of relational insecurity, self-evaluation, and demographic characteristics rather than through the independent effect of a single variable.

Among available analytical approaches, C&RT was preferred because it offers a balance between data-driven flexibility and interpretability that the alternatives outlined in the Introduction (regression, latent profile analysis, random forests) do not jointly provide. This balance is particularly suited to psychological data, in which variables such as attachment styles and self-esteem may operate differently across demographic and relational profiles.

Among decision tree approaches, C&RT was preferred because it produces binary recursive splits that are directly applicable to classification problems and easy to interpret in applied psychological research. Since manipulation tendency scores were categorized into low and high groups in the present study, C&RT provided a suitable framework for identifying the sequence of variables that most effectively differentiated these two groups. Unlike traditional regression-based models, C&RT does not require the assumption that relationships between predictors and the outcome are linear or additive, and it can reveal interaction-like patterns by identifying different splitting rules across subgroups (Breiman et al., 1984; Hastie et al., 2009; Song & Lu, 2015). This characteristic is particularly relevant for psychological data, where variables such as attachment styles and self-esteem may operate differently depending on individuals’ demographic and relational profiles. Decision tree methods have increasingly been used in psychological and behavioral research because they provide interpretable models capable of identifying complex and subgroup-specific patterns among variables.

Accordingly, the C&RT model was used as an exploratory and interpretable classification tool rather than as a diagnostic or causal model. The resulting tree structure was expected to show which psychological and demographic variables entered the classification process first, how subsequent splits refined the classification of manipulation tendency levels, and which combinations of characteristics formed distinct terminal profiles. Thus, the use of C&RT was consistent with the aim of describing the multifactorial and hierarchical organization of manipulation tendencies among adults, while avoiding causal claims beyond the scope of the cross-sectional research design.

## 2. Method

### 2.1. Research Model

This study used a cross-sectional design to examine factors associated with adults’ relationships among psychological and demographic variables and to describe how different combinations of these variables classify individuals with varying levels of manipulation tendency. In line with a correlational survey framework, the study aimed to characterize the pattern of associations among variables (Creswell & Creswell, 2018). A decision tree classification approach based on the C&RT algorithm was applied to model the hierarchical.

### 2.2. Study Group

A total of 601 adults were recruited. Prior to the main analyses, the dataset was screened to ensure data quality by checking missing data, inattentive responding, and outliers. Participants were recruited via convenience and snowball sampling methods through online platforms and social media announcements. The inclusion criteria were being over 18 years of age and volunteering to participate. Regarding the demographic background, the majority of the sample consisted of university students and young professionals from middle-class socioeconomic backgrounds. No clinical diagnosis was screened; thus, the sample represents a general community population. Participants were excluded if they had incomplete responses on key measures, showed indicators of careless responding (e.g., repetitive response), or exhibited extreme values on primary variables identified using Tukey’s 1.5 × IQR criterion. After these procedures, the final sample consisted of 543 adults living in Turkey (358 women, 65.93%; 185 men, 34.07%). Participants were aged 18–45 years (M = 25.79, SD = 4.23). Data were collected in 2024 via an online survey administered through Google Forms. All participants provided informed consent, and the study procedures were approved by the relevant institutional ethics committee as stated in the Ethical Statement section.

### 2.3. Data Collection Tools

#### 2.3.1. Personal Information Form

A brief personal information form was used to collect basic demographic data from participants. Specifically, participants reported their gender and age.

#### 2.3.2. Manipulation Scale in Human Relations

The Manipulation Scale in Human Relations, developed by Yılmaz (2018), assesses individuals’ tendencies to use manipulative behaviors in interpersonal relationships. The scale is a 39-item instrument rated on a 5-point Likert scale and comprises five subdimensions: aggressive manipulation, emotional manipulation, victim selection, self-concealment, and strategy use. In the scale development study, internal consistency was reported to be high for all subdimensions (Cronbach’s alpha = 0.92–0.95), and acceptable for the total score (alpha = 0.90). Confirmatory factor analysis supported the five-factor structure and indicated acceptable model fit (X^2^/df = 2.16, NFI = 0.93, TLI = 0.91, IFI = 0.93, RMSEA = 0.05, GFI = 0.93, AGFI = 0.90). In the present study, internal consistency reliability was re-examined for the current sample. The total score of the Manipulation Scale in Human Relations showed high internal consistency (Cronbach’s alpha = 0.92), indicating that the items reliably measured the same underlying construct in this dataset. In this study, the total score of the Manipulation Scale in Human Relations was preferred as the dependent variable. This approach is justified by the scale’s high internal consistency and its conceptual structure, where sub-dimensions collectively represent a single higher-order construct of manipulative tendency. According to psychometric theory, when subscales are highly intercorrelated and reflect a common underlying factor, using a composite total score provides a more parsimonious and reliable estimate of the target construct than analyzing individual components separately (Nunnally & Bernstein, 1994). By focusing on the total score, the model captures the cumulative effect of manipulative behaviors, which is more aligned with the study’s aim of identifying general risk profiles. In the present study, the total composite score (39 items) of the Manipulation Scale in Human Relations was used as the outcome variable; this composite reflects general manipulative tendency.

#### 2.3.3. Relationship Scales Questionnaire

Attachment styles were assessed using the Relationship Scales Questionnaire, originally developed by Griffin and Bartholomew (1994) and adapted into Turkish by Sümer and Güngör (1999). The RSQ consists of 17 items measuring four attachment styles: secure, dismissive, fearful, and preoccupied. Participants rated how well each statement described them on a 7-point Likert scale ranging from 1 (does not describe me at all) to 7 (describes me completely). Secure and dismissive attachment were each assessed with five items, whereas preoccupied and fearful attachment were assessed with four items each. Subscale scores were computed by averaging item responses within each attachment style. Following the common scoring approach for the RSQ, participants can be classified into the attachment style for which they obtain the highest subscale score. Previous research has noted that RSQ subscales may show relatively low internal consistency, while demonstrating acceptable test–retest reliability. In the Turkish adaptation study, a four-factor structure was supported, and test–retest reliability coefficients ranged from 0.54 to 0.61 across the four dimensions (Sümer & Güngör, 1999). In the present study, internal consistency reliability (Cronbach’s alpha) for the subscales was 0.60 for secure attachment, 0.65 for dismissive attachment, 0.62 for fearful attachment, and 0.66 for preoccupied attachment. Consistent with prior RSQ research, these alpha coefficients were modest, which may be expected given the brief length of the subscales and the heterogeneous content of attachment-related items.

#### 2.3.4. Rosenberg Self-Esteem Scale

Self-esteem was assessed using the Rosenberg Self-Esteem Scale, originally developed by Rosenberg (1965) and adapted into Turkish by Cuhadaroglu (1986). Although the broader Rosenberg inventory includes multiple subdimensions, only the self-esteem subscale was used in the present study. The self-esteem subscale consists of 10 items rated on a 4-point Likert scale ranging from 1 (strongly agree) to 4 (strongly disagree). Items 1, 2, 4, 6, and 7 are reverse-coded. Total scores range from 10 to 40, with higher scores indicating higher self-esteem. Previous research reported adequate reliability for the original scale (internal consistency = 0.80; test–retest = 0.85) and for the Turkish adaptation (internal consistency = 0.71; test–retest = 0.75) (Cuhadaroglu, 1986). In this study, reliability was re-estimated for the current dataset, and the 10-item self-esteem subscale yielded a Cronbach’s alpha of 0.88, indicating high internal consistency.

### 2.4. Data Analysis

The analysis examined factors associated with manipulation tendency using decision tree modeling to explore the relational patterns among variables. Manipulation tendency was represented in a hierarchical structure, and the most informative splits at each node were identified by maximizing the reduction in Gini impurity rather than through formal statistical significance testing. This approach clarified the relative contribution of each variable to the classification structure within the recursive partitioning framework.

To create the binary outcome variable required for classification-based C&RT analysis, the total composite manipulation tendency score (sum of all 39 items across the five subdimensions) was dichotomized using the sample median as the empirical cut-off point. Participants whose manipulation tendency scores were below the median were classified into the low manipulation tendency group, whereas participants whose scores were equal to or above the median were classified into the high manipulation tendency group. The median was selected as the cut-off because it is a distribution-based, sample-specific threshold that does not depend on arbitrary or theoretically unjustified score values, and because a binary outcome is required by the C&RT algorithm, which performs recursive binary partitioning based on class labels (Breiman et al., 1984; Loh, 2011). In the absence of an established clinical or normative cut-off score for manipulation tendency in adult community samples, a median split provides a transparent and replicable empirical criterion that yields approximately equal-sized comparison groups, which is methodologically desirable for tree-based classification because it reduces class imbalance and improves the stability of split-point estimates (Song & Lu, 2015). Median-based dichotomization has also been used and defended in prior psychological and behavioural science research as a practical and interpretable strategy when the analytic goal is profile identification or group-level classification rather than estimation of continuous effect sizes (Farrington & Loeber, 2000; DeCoster et al., 2011; Iacobucci et al., 2015). This median-based classification was considered appropriate for the exploratory and classification-oriented purpose of the study because it enabled the continuous manipulation tendency score to be transformed into a binary outcome suitable for C&RT analysis and helped maintain relatively balanced comparison groups for model training and testing. However, the median split was not intended to represent a clinical, diagnostic, or theoretically established threshold. Rather, it was used as an empirical grouping strategy to identify psychological and demographic profiles associated with comparatively lower and higher manipulation tendency levels. It should also be noted that, although the dichotomized variable was used as the outcome, all continuous predictors (self-esteem, the four attachment subscale scores, and age) were entered into the C&RT model in their original continuous form, which preserves within-predictor variability and mitigates concerns about loss of information at the predictor level. Nevertheless, dichotomization of continuous variables may reduce statistical power and obscure variability within groups (Altman & Royston, 2006), and therefore the findings should be interpreted within the exploratory and classification-oriented scope of the present study.

#### 2.4.1. Model Specifications and Stopping Rules

In this study, a Classification and Regression Tree (C&RT) model was developed to examine the factors most strongly associated with manipulation tendencies in adults. The C&RT algorithm consists of three steps: creation of the maximum tree, tree pruning, and selection of the optimal tree. The C&RT algorithm creates the classification tree by applying binary splitting of attributes to right and left nodes based on class labels (Breiman et al., 1984; Loh, 2011). To ensure reproducibility of the analyses, a fixed random seed value of 123 was used prior to data partitioning, The dataset (*N* = 543) was randomly partitioned into training (70%, *n* = 381) and testing (30%, *n* = 162) subsets using stratified random sampling to maintain the distribution of the outcome variable across both sets. The training dataset was used for model development and internal validation procedures, whereas the independent testing dataset was reserved exclusively for final model evaluation. Several stopping rules were implemented to optimize model performance and prevent overfitting. Specifically, a minimum of 10 cases was required for a parent node to be eligible for splitting (minsplit = 10), while each child node was required to contain at least 10 cases (minbucket = 10) to ensure statistical reliability and prevent the creation of overly specific terminal nodes. The complexity parameter (cp) was set to 0.001 to allow more detailed subgroup partitioning while controlling excessive growth through additional stopping rules. The maximum tree depth was set to 5 levels, though the final pruned tree was considerably shallower to maintain interpretability. The Gini impurity index was employed as the splitting criterion to identify the optimal splits at each node, measuring the probability of misclassification at each potential split point. Following initial tree growth, cost-complexity pruning was applied to determine a tree size that balanced model complexity and fit. The 10-fold cross-validation (xval = 10) was performed exclusively on the training set (n = 381) using the rpart package’s internal cross-validation procedure; the test set (n = 162) was held out throughout this process and used only for final performance evaluation after pruning was complete. The optimal complexity parameter (cp) was selected using the minimum cross-validated error (min xerror) rule, which identifies the cp value yielding the lowest cross-validated misclassification error, and this cp value was used to prune the maximum tree to its final, parsimonious form.

#### 2.4.2. Model Performance Evaluation

In this study, performance evaluation criteria based on binary classification were used for model performance evaluation measurement. These criteria were determined as accuracy, sensitivity, specificity, precision, and F-score. The classification to be used for the criteria and the calculation of the criteria are given in Table 1.

“Sensitivity” is the probability that the predicted model is also positive (1) when the observed model is positive (1), while “specificity” is the probability that the predicted model is also negative (0) when the observed model is negative (0). In the constructed cross-table, both sensitivity and specificity probabilities are expected to be high simultaneously. With the ROC curve, the plotting of the model’s sensitivity against the model’s (1-specificity) ratio at different cut-off points is obtained. As done in every classification process, methods deal with establishing the balance between sensitivity and specificity. The area under the ROC curve can be defined as AUC (area under curve), and this AUC is accepted as the best indicator of the model’s success in distinguishing positives from negatives. When this area is 1, it means that positives are perfectly separated from negatives.

All analyses were conducted using R statistical software (version 4.4.3; R Core Team, 2025) with the rpart package (version 4.1.24; Therneau & Atkinson, 2025) for tree construction, the caret package (version 7.0.1; Kuhn, 2024) for model validation and performance evaluation, the rpart.plot package (version 3.1.4; Milborrow, 2024) for tree visualization, and the pROC package (version 1.19.0.1; Robin et al., 2024) for ROC curve analysis and AUC calculation.

## 3. Results

### 3.1. Descriptive Statistics

Descriptive statistics were computed to characterize the sample and examine the distribution of key study variables. The final sample comprised 543 participants with a mean age of 25.79 years (SD = 4.23; range = 18–45 years). The primary outcome variable, manipulation tendency, yielded a mean score of 117.43 (SD = 18.94; range = 62–165). The attachment dimensions yielded the following mean scores: preoccupied attachment (M = 11.96, SD = 2.51; range = 5–18), fearful attachment (M = 16.00, SD = 2.28; range = 9–22), secure attachment (M = 21.74, SD = 6.30; range = 7–35), and dismissive attachment (M = 20.01, SD = 2.82; range = 12–29). Self-esteem scores exhibited a mean of 24.17 (SD = 4.44; range = 11–39). The gender distribution of the sample consisted of 358 women (65.9%) and 185 men (34.1%).

### 3.2. Classification and Regression Tree (C&RT) Analysis

In this study, a decision tree model was constructed to examine the hierarchical structure of variables involved in the classification of manipulation tendency levels. The final model included nine terminal nodes based on the training sample of 381 participants and classified individuals into higher and lower manipulation tendency groups through hierarchical splits involving attachment styles, self-esteem, age, and gender.

**Figure 1:**
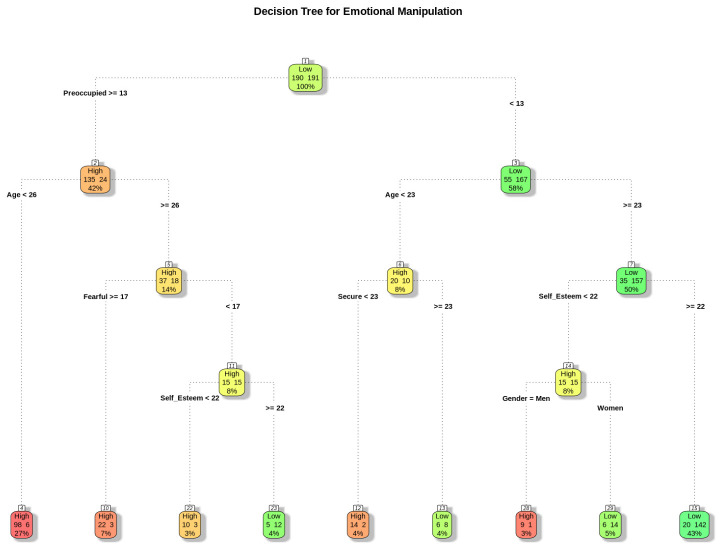
Decision Tree for Manipulation Tendency. NOTE: In the decision tree visualization, each node contains three key pieces of information. At the top of each node is the predicted manipulation tendency level (“High” or “Low”). In the middle, a two-number distribution is presented, where the first number indicates the number of individuals observed in the high manipulation tendency group, and the second number indicates the number of individuals observed in the low manipulation tendency group. At the bottom of each node, the percentage of participants represented by that node is reported. For instance, a node displaying “High/98—6/27%” indicates that, within this profile, 98 individuals are observed in the high manipulation tendency group, 6 individuals are observed in the low manipulation tendency group, and 27% of the total sample falls into this category. The conditions shown on the branches between nodes (e.g., “Preoccupied < 13”) indicate the splitting criteria used by the model. The color shading reflects the relative concentration of the predicted outcome within each node, with darker red tones indicating a higher proportion of high manipulation tendency cases and green tones indicating a higher proportion of low manipulation tendency cases.

The final pruned C&RT model was developed on the training sample (N = 381) and produced nine terminal nodes. At the root, the two outcome categories were nearly balanced (high = 190; low = 191), so no class-imbalance correction was required. The first partition was based on preoccupied attachment at a threshold of 13, which divided the sample into two structurally distinct branches that organized all subsequent splits. The detailed counts and percentages associated with each node are presented in Figure 1; the interpretation below focuses on the classification profiles produced by the hierarchical structure rather than on a node-by-node restatement of those values.

Within the high-preoccupied branch (preoccupied ≥ 13), age operated as the secondary partitioning variable (threshold = 26 years). Younger participants in this branch were almost uniformly assigned to the high manipulation tendency category, forming the most consistently classified profile in the tree. For older participants within the same branch, fearful attachment introduced an additional layer of differentiation: higher fearful attachment scores were associated with continued classification into the high category, whereas lower fearful attachment scores produced a more mixed pattern, within which self-esteem provided a further refinement, with lower self-esteem aligning with the high category and higher self-esteem with the low category. Within the low-preoccupied branch (preoccupied < 13), age again served as the secondary partition (threshold = 23 years), and the structure was completed by self-esteem and, in one terminal pathway, gender. The majority of older participants with adequate self-esteem (≥22) were classified into the low manipulation tendency category, representing the most clearly differentiated low-category profile. Younger participants in this branch showed more heterogeneous outcomes, with secure attachment providing additional differentiation. Within the small subgroup of older participants with low self-esteem, a gender-based partition emerged in a single terminal pathway; given the limited number of cases in this cell, this conditional pattern is reported descriptively.

Taken together, three broad classification profiles are produced by the hierarchical structure of the model. The first profile—younger adults with elevated preoccupied attachment—corresponds to the most consistent classification into the high manipulation tendency category. The second profile—older adults with low preoccupied attachment and adequate self-esteem—corresponds to the most consistent classification into the low category. The remaining terminal nodes represent intermediate, conditional profiles in which self-esteem, fearful or secure attachment, and (in one narrow pathway) gender further refine classification. These pathways describe how the included variables jointly organize manipulation tendency classifications within the present sample and should be interpreted as conditional, sample-specific patterns derived from recursive partitioning rather than as estimates of population prevalence or causal effects.

In C&RT models, variable importance is computed based on the total reduction in node impurity (Gini index) attributable to each predictor across the entire tree structure. The variable importance rankings derived from the decision tree model are summarized in Table 2.

Preoccupied attachment style exhibited the highest importance score (68.61), indicating that it was the variable most frequently involved in the hierarchical splitting process of the model. This pattern indicates that preoccupied attachment was the primary splitting variable within the decision tree structure.

Self-esteem ranked second with an importance score of 34.76, followed closely by age, which ranked third with an importance score of 34.33. These importance values indicate that self-esteem and age were similarly involved in the classification process across multiple branches of the tree, although their contributions were conditional on specific node-level splits. Within the context of decision tree modeling, these rankings reflect the relative contribution of variables to the model’s classification structure rather than the magnitude of their effects or causal influence.

The dismissive attachment style ranked fourth in the variable importance analysis (importance score = 28.48), indicating that it contributed to the classification structure of the decision tree across several branches.

Although dismissive attachment was ranked fourth in variable importance, it does not appear as a primary split in the final decision tree. In C&RT models, variable importance values may reflect not only primary splitting variables but also variables that contribute indirectly to impurity reduction across alternative or lower-level partitions. Therefore, dismissive attachment may have contributed to the classification structure by capturing variance shared with other attachment-related variables, even though it was not selected as a dominant primary split in the final pruned tree. Gender followed as the fifth most frequently utilized variable, with an importance score of 21.58, reflecting its contribution within specific decision pathways. The fearful attachment style ranked sixth, with an importance score of 18.27, indicating comparatively lower involvement in the splitting structure.

Secure attachment exhibited the lowest importance score (4.11), indicating that it was least frequently used in the hierarchical splitting process. Within the decision tree framework, this low importance suggests that secure attachment contributed minimally to the differentiation of individuals into higher versus lower manipulation tendency groups and was primarily involved in a small number of terminal classification paths.

Overall, the distribution of variable importance values reflects a multifactorial classification structure underlying manipulation tendencies. Preoccupied attachment emerged as the most prominent classifier within the model, while self-esteem and age together constituted a substantial portion of the remaining classification structure. The other variables—dismissive attachment, gender, fearful attachment, and secure attachment—contributed incrementally within specific conditional pathways, highlighting the hierarchical and interaction-based nature of the decision tree model rather than the dominance of any single predictor.

To allow transparent inspection of classification performance beyond aggregated metrics, the full confusion matrices for both sets are reported here. In the training set (n = 381), of the 190 participants in the high manipulation tendency group, 154 were correctly classified as high (true positives) and 36 were misclassified as low (false negatives); of the 191 participants in the low group, 176 were correctly classified as low (true negatives) and 15 were misclassified as high (false positives). In the test set (n = 162), of the 81 participants in the high group, 60 were correctly classified as high and 21 were misclassified as low; of the 81 participants in the low group, 71 were correctly classified as low and 10 were misclassified as high. These distributions correspond to the aggregated metrics reported in Table 3.

The developed decision tree model demonstrated satisfactory classification performance in distinguishing manipulation tendency levels, achieving an accuracy of 86% in the training set (n = 381) and 81% in the test set (n = 162). Sensitivity values were 0.81 for the training set and 0.74 for the test set, indicating that the model successfully classified a substantial proportion of individuals in the high manipulation tendency category. Specificity values were 0.92 and 0.88, respectively, suggesting that individuals in the low manipulation tendency category were classified with high accuracy.

The difference in accuracy between the training and test sets was 5%, and the difference in F1-scores was 0.06, suggesting limited performance decline when applied to unseen data. In addition, the model yielded high precision values (training = 0.91, test = 0.86) and balanced accuracy rates (87% and 81%, respectively), suggesting relatively balanced classification performance across outcome categories.

Overall, these findings indicate that the decision tree model—optimized using 10-fold cross-validation and predefined model constraints (cp = 0.001, minbucket = 10, maxdepth = 5)—provides an exploratory and interpretable classification framework for differentiating manipulation tendency profiles. The model should not be interpreted as a diagnostic tool but rather as an exploratory classification framework for examining how psychological and demographic variables are hierarchically organized within distinct classification pathways.

**Figure 2:**
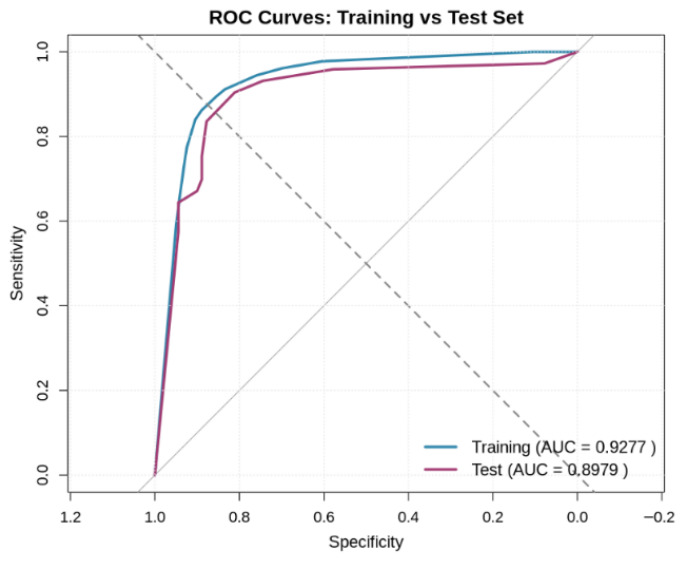
ROC Curves: Training and Test Set.

The ROC curve analysis presented in Figure 2 evaluates the classification performance of the decision tree model in distinguishing high and low manipulation tendency groups across the training and test sets. The area under the curve (AUC) value for the training set was 0.93, indicating excellent discriminative ability in differentiating between the two outcome categories. In the test set, the AUC value was 0.90, suggesting that the model maintains high discriminative performance when applied to previously unseen data.

The small difference between the training and test AUC values (ΔAUC = 0.03) suggests exploratory discriminative performance across datasets and demonstrates acceptable generalization performance. Furthermore, the close proximity of both ROC curves to the upper-left corner reflects an appropriate trade-off between sensitivity and specificity across classification thresholds, indicating that the model effectively distinguishes between high and low manipulation tendency classifications.

The fact that both AUC values exceed 0.90 indicates that the decision tree model substantially exceeds chance-level discrimination (AUC = 0.50) across both datasets. Overall, the ROC analysis supports the model’s discriminative capacity within the present sample as a classification tool for identifying manipulation tendency profiles. Consistent with the decision tree methodology, these results reflect classification-based differentiation rather than linear, correlational, or causal relationships between the included variables.

## 4. Discussion

This study examines the psychological and demographic factors underlying manipulation tendencies in adults through a comprehensive Classification and Regression Tree (C&RT) analysis. The hierarchical structure of the model illustrates how variables such as attachment styles, self-esteem, gender, and age interact in shaping manipulation tendencies within specific relational and developmental contexts. The developed model demonstrated acceptable classification performance across both the training and test sets within the present sample. However, since the model has not been validated on an independent external dataset, the stability of the resulting tree structure should be interpreted cautiously.

When the findings were evaluated across the full sample, the variables that emerged as the most influential classifiers of manipulation tendency within the decision tree model were preoccupied attachment style, self-esteem, age, dismissive attachment style, gender, fearful attachment style, and secure attachment style, respectively. Importantly, beyond identifying influential variables, the decision tree framework provides conditional and profile-based insights into how combinations of psychological and demographic characteristics jointly differentiate individuals with respect to manipulation tendencies.

Rather than estimating average linear associations, the C&RT approach reveals distinct classification pathways by specifying the conditions under which individuals are more likely to be classified into higher or lower manipulation tendency profiles. This person-centered and interactional perspective allows manipulation tendency to be conceptualized not as a uniform trait or behavior, but as a context-dependent tendency that emerges through the dynamic interplay of attachment-related patterns, self-evaluative processes, and developmental factors.

The findings indicate that preoccupied attachment style emerged as the most salient splitting variable within the decision tree model, differentiating individuals classified into higher versus lower imply a direct or linear relationship; rather, it highlights the central role of preoccupied attachment in combination with other variables such as age, self-esteem, and gender in shaping classification outcomes manipulation tendencies across specific conditional pathways. Importantly, within the C&RT framework, this finding does not.

According to Bartholomew and Horowitz’s (1991) four-category attachment model, individuals with a preoccupied attachment style are characterized by negative internal working models of the self and positive internal working models of others. This configuration is associated with excessive dependency, heightened approval-seeking, and a pronounced fear of abandonment in close relationships. Mikulincer and Shaver (2007) describe preoccupied attachment as involving hyperactivation strategies, in which chronic activation of the attachment system motivates intense efforts to maintain proximity and obtain reassurance. Although the present cross-sectional, classification-based design does not allow direct testing of the motivational mechanisms underlying preoccupied attachment, this theoretical framework offers one plausible interpretation for why elevated preoccupied attachment scores co-occur with higher manipulation tendency classifications in the model. The data from the present study speak only to this co-occurrence; the question of whether manipulative tendencies actually function to preserve relational closeness in individuals high in preoccupied attachment remains an empirical question for future, design-appropriate research.

Similarly, Cassidy and Berlin (1994) noted that individuals with preoccupied attachment may engage in persistent attention-seeking behaviors, emotional dependency, and guilt induction to sustain their partners’ involvement. Although empirical studies directly examining the link between preoccupied attachment and emotional manipulation remain limited, Schotman (2022) provides direct evidence, showing that preoccupied attachment significantly differentiates individuals with higher levels of manipulation tendencies. Complementing this evidence, Wei et al. (2005) reported that preoccupied attachment is associated with lower relationship satisfaction and elevated relational stress. Whether elevated relational stress is then regulated through manipulative interpersonal strategies, however, was not assessed in the present study; this proposition is offered only as a candidate explanation for the co-occurrence pattern observed in the tree and remains to be tested with designs capable of identifying such regulatory processes.

Feeney (1999) further emphasized that individuals with preoccupied attachment experience difficulties in emotional regulation and a heightened need for control within relationships. Consequently, whether consciously or unconsciously, they may rely on strategies such as guilt induction, exaggerated emotional expression, emotional blackmail, or self-victimization to secure reassurance and maintain relational bonds (D. G. Dutton & Painter, 1993; Knox et al., 2023). Additionally, behaviors such as excessive monitoring or attempts to restrict a partner’s social environment can serve as anxiety-driven regulatory strategies aimed at reducing perceived threats of abandonment.

Taken together, and consistent with both attachment theory and emerging empirical evidence, the findings of the current study indicate that preoccupied attachment occupies a central position within the hierarchical structure of manipulation tendency classifications. Rather than representing a universal vulnerability, preoccupied attachment appears to function as a key contextual factor that, in interaction with developmental and self-esteem variables, differentiates individuals who are more likely to exhibit elevated manipulation tendencies.

It should be noted that the subscales of the Relationship Scales Questionnaire exhibited relatively low internal consistency (alpha values between 0.60 and 0.66). These values may introduce noise into the model, potentially affecting the stability of the tree splits and the ranking of variable importance. Consequently, the findings regarding attachment patterns should be interpreted with caution, as measurement error associated with low reliability could distort the hierarchical structure of the classification tree.

Self-esteem also functioned as a discriminating variable in the decision tree, contributing to the differentiation of manipulation tendency classifications within specific conditional pathways rather than as a linear, main-effect predictor. Within the C&RT framework, this result reflects the role of self-esteem in differentiating individuals classified into higher versus lower manipulation tendency profiles under specific conditional pathways, rather than indicating a direct or linear association. The conceptual question this raises is why self-esteem resources should matter for manipulation tendencies at all, and how they intersect with attachment-related vulnerability.

Self-esteem is conceptualized as a multidimensional construct reflecting individuals’ perceptions of themselves as valuable, acceptable, and lovable (Harter, 1999). Although empirical studies directly examining the relationship between self-esteem and manipulation tendencies remain limited, several lines of indirect evidence are noteworthy. For example, Kurtyılmaz et al. (2017) demonstrated that self-esteem influences relational aggression through social anxiety. Because manipulation strategies are often considered core components of relational aggression, this finding may be interpreted as an indirect indicator of self-esteem’s role in manipulative interpersonal dynamics. Complementing this perspective, Grieve and Panebianco (2013) reported a significant association between aggression-related traits and manipulation tendencies.

Moreover, previous research suggests that adults with low self-esteem, heightened dependency needs, or insecure attachment patterns may be particularly vulnerable to maladaptive relationship dynamics, including manipulative interpersonal strategies (Braiker, 2004; Mruk, 2006). In this context, the present findings indicate that lower self-esteem may act as a contextual vulnerability factor, increasing the likelihood of individuals being classified into profiles characterized by higher manipulation tendencies under certain relational and developmental conditions.

Furthermore, tendencies toward manipulation have frequently been studied in relation to personality traits associated with the Dark Triad—narcissism, Machiavellianism, and psychopathy (Austin et al., 2007). The complex relationships between self-esteem and personality traits, particularly narcissism and Machiavellianism, are noteworthy in individuals exhibiting manipulative behavior. Individuals with narcissistic traits often display an inflated self-image, which may, paradoxically, be rooted in fragile or low self-esteem. Narcissistic individuals may also engage in manipulative tactics to enhance social status and elicit admiration from others (Austin et al., 2007). Within this theoretical and empirical framework, the emergence of self-esteem as a key distinguishing variable in the present decision tree model represents a significant and theoretically coherent finding.

Age also functioned as a conditional splitting variable in the model. The directional pattern observed in the tree—younger participants more often falling into higher manipulation tendency profiles—converges with previous reports that emerging and early-young adults score higher on manipulation tendency than older adult subgroups (Tekiner, 2022; Şenyuva & Yavuz, 2024). Beyond replicating this descriptive pattern, the more substantive question is what developmental processes might make younger adulthood a period of heightened reliance on manipulative interpersonal strategies.

Two complementary developmental considerations are particularly relevant. First, emerging and early-young adulthood is characterised by ongoing consolidation of identity, autonomy, and intimacy capacities, and by relatively unstable romantic relationships in which negotiation of closeness and control is salient (Arnett, 2000; Mikulincer & Shaver, 2016); under these conditions, attachment-related anxieties may be more readily channelled into interpersonally controlling behaviours. Second, capacities relevant to alternative, non-manipulative regulation—such as effective emotion regulation, perspective-taking, and constructive conflict resolution—continue to develop into the late twenties and thirties (Aftab & Malik, 2021), which is consistent with the age thresholds (23 and 26 years) observed in the present tree. The fact that the literature is not uniform on this point—e.g., Esin (2022) reported peak manipulation levels in the 34–41 age range—underscores that age in our study is best understood as a contextual marker for these developing competencies rather than as a chronological cause in itself, and that the specific age cut-points obtained here are sample-dependent.

Aftab and Malik (2021) suggested that although manipulation tendencies may appear at similar levels during adolescence and young adulthood, the expression and consequences of such behaviors differ across developmental stages. As emotional regulation skills, social awareness, and interpersonal competencies mature with age, the likelihood of relying on manipulative interpersonal strategies may decrease.

Dismissive attachment style emerged as the fourth discriminating variable within the decision tree model. Rather than indicating a direct or linear effect, this finding suggests that dismissive attachment contributes to the classification of manipulation tendencies within specific conditional pathways involving other psychological and demographic variables. Dismissive attachment is conceptualized as a form of avoidant attachment characterized by an excessive emphasis on autonomy and a defensive denial of the need for closeness and dependence in interpersonal relationships (Bartholomew & Horowitz, 1991).

When the relevant literature is examined, empirical studies directly linking attachment styles to manipulation are notably limited. One of the few studies addressing this association is Schotman (2022), who examined lying and emotional manipulation in relation to attachment styles and found that avoidant attachment was significantly associated with higher levels of both lying and manipulation. This finding provides empirical support for the classification patterns observed in the present study.

According to Mikulincer and Shaver (2007), individuals with avoidant attachment experience discomfort with emotional intimacy and dependence. As a result, they employ strategies to maintain emotional distance in relationships, such as ignoring a partner’s emotional needs, avoiding emotional sharing, or physically distancing themselves. These behaviors, whether conscious or unconscious, can exert a manipulative effect on the partner’s emotional responses. Moreover, individuals with avoidant attachment tend to avoid direct conflict; instead of confronting issues, they may express anger or dissatisfaction in passive-aggressive ways, including sarcastic comments, shrugging, or refusing to communicate (Pistole, 1989; Hazan & Shaver, 1987). Notably, the use of “silent treatment” serves as a manipulative tool, generating anxiety, guilt, or helplessness in their partners to punish them, achieve compliance, or steer an argument in their favor (Bowlby, 1969; Tamara, 2023). From this perspective, it is notable that avoidant attachment is associated with higher classification into manipulation tendency profiles.

From a decision tree perspective, the emergence of dismissive attachment as a discriminating variable suggests that avoidant relational strategies may contribute to higher manipulation tendency classifications under particular combinations of attachment insecurity, self-esteem, age, and gender. Thus, dismissive attachment appears to play a meaningful contextual role in the hierarchical differentiation of manipulation tendencies rather than representing a generalized or uniform vulnerability.

Gender ranked fifth in variable importance and, critically, did not function as a uniform predictor across the tree. It appeared as a splitting variable in only one terminal pathway—the low-preoccupied/older/low-self-esteem branch. The present results therefore do not support a generalised claim that men, as a group, are more inclined toward manipulation tendency; they indicate only that, within this very specific combination of low preoccupied attachment, older age, and low self-esteem, being male was associated with a higher classification probability. Any broader interpretation of male versus female tendencies must be drawn from the wider literature rather than from this single conditional split.

This classification pattern aligns with a substantial body of empirical research reporting higher levels of manipulation tendencies or manipulativity-related traits among men (Grieve & Panebianco, 2013; Brewer & Abell, 2017; Esin, 2022; Tekiner, 2022; Şenyuva & Yavuz, 2024). Hyde et al. (2021) found that women are significantly less likely than men to engage in both malicious and insincere manipulation, suggesting that such behaviors are particularly prevalent in traditional societies that valorize stereotypical male traits, such as assertiveness and independence. Similarly, Hyde and Grieve (2014), in a study examining participants’ beliefs about their ability to manipulate others and the frequency of such behaviors, reported that men scored higher on both measures.

Cultural and gender-role variables, such as endorsement of hegemonic or traditional masculinity ideologies, have been proposed in the broader literature as relevant to manipulative behaviour in both men and women (Waddell et al., 2020). However, the present study did not measure masculinity ideology, gender-role endorsement, or any related cultural construct, and the conditional gender split observed here cannot be interpreted as evidence for or against such mechanisms. Examining whether gender-role and cultural variables account for the conditional gender pattern observed in this tree is a question for designs that explicitly include such measures.

In the decision tree analysis, the male/low-self-esteem cell within this older, low-preoccupied branch showed the highest concentration of high-manipulation classifications. Given the very small cell size, this pattern should be regarded as preliminary and as in need of replication; it is offered as a tentatively suggestive result rather than a confirmatory finding. With this caveat in mind, it is consistent with theoretical accounts proposing that low self-esteem can motivate compensatory or self-protective interpersonal strategies (Baumeister et al., 1996), although such mechanisms were not directly tested in the present study.

Secure and fearful attachment styles emerged as additional discriminating variables within the decision tree, although both contributed only within narrowly defined branches and with low overall variable importance. Empirical studies that directly examine the link between these specific attachment styles and manipulation tendency remain scarce; one of the few available studies (Schotman, 2022) reported that avoidant attachment, which conceptually overlaps with fearful attachment, is significantly associated with manipulation tendency. Fearful attachment is characterised by simultaneous desire for and avoidance of closeness, producing contradictory and inconsistent relational behaviour (Main & Solomon, 1990); within the present tree, it appeared only in the high-preoccupied/older subgroup, suggesting that its contribution to manipulation classification is conditional rather than general.

In this respect, the finding that secure attachment style emerged as a discriminating variable of manipulation tendency should be interpreted cautiously. As discussed earlier, the internal consistency of the secure attachment subscale was relatively low, which may have affected the stability and reliability of this result. Moreover, within a decision tree framework, the emergence of a variable does not necessarily indicate a direct or dominant effect. Rather, it reflects the variable’s role within specific conditional pathways formed by interactions with other predictors. In this context, secure attachment may function as a differentiating variable only under particular combinations of self-esteem, age, or gender, rather than representing a general vulnerability to manipulation tendency. Therefore, this finding should not be interpreted as contradicting existing attachment literature, but as a context-dependent result specific to the hierarchical structure of the model. Specifically, the very low variable importance of secure attachment (importance score = 4.11; relative contribution = 2.0%) coincides with the lowest reliability among the four RSQ subscales (α = 0.60). This combination suggests that the marginal role of secure attachment in the present tree may partly reflect measurement error rather than a genuinely weak conceptual contribution; the same caution, although to a lesser degree, applies to the remaining attachment subscales, all of which fell below the conventional 0.70 threshold and may have attenuated their estimated importance and the precise positioning of their splits within the tree. From a theoretical perspective, secure attachment is generally associated with effective emotional regulation, autonomy, and adaptive interpersonal functioning, which are typically considered protective factors against manipulative relationship dynamics (Mikulincer & Shaver, 2016; Fraley & Roisman, 2019). However, emerging research suggests that securely attached individuals may still engage in strategic interpersonal behaviors depending on contextual demands and relational goals, particularly in situations involving power negotiation or boundary setting (Overall & Simpson, 2015; Simpson & Rholes, 2015).

## 5. Conclusions

In conclusion, this study demonstrates how levels of manipulation tendency in adulthood are differentiated by and associated with the combined contribution of demographic variables (age and gender) and psychological characteristics (attachment styles and self-esteem) using a comprehensive decision tree analysis. Within this hierarchical structure, preoccupied attachment style emerged as the most salient discriminating variable, particularly among younger adults, suggesting that individuals with elevated preoccupied attachment scores tend to be classified into higher levels of manipulation tendencies.

The prominent role of preoccupied attachment underscores the importance of relational schemas and attachment-related anxieties in the manifestation of manipulative tendencies. At the same time, the contributions of self-esteem, age, and secure attachment provide a more nuanced and integrative understanding of manipulation tendency. Specifically, age-related differences observed in the model suggest that participants in different age ranges are classified differently with respect to attachment-related profiles, although the present cross-sectional, classification-based design does not permit inferences about developmental mechanisms. Additionally, the gender-specific patterns identified in conjunction with low self-esteem point to the relevance of considering individual differences when interpreting manipulation tendencies.

Because the present study is cross-sectional and based on classification rather than causal modelling, these findings should not be read as direct clinical prescriptions. They may, however, be cautiously informative for hypothesis generation in applied settings: the prominence of preoccupied attachment and self-esteem in the model is consistent with broader literature in which these constructs are addressed in psychological support work with young adults, and our results may help refine which sub-profiles warrant closer empirical attention in future intervention research. Overall, rather than pointing to a single causal pathway, the results highlight the interactive role of attachment, developmental factors, and psychological characteristics in manipulation tendencies.

## 6. Recommendations

Because the present design is cross-sectional and classification-based, the following recommendations are framed as directions for future research rather than as clinical, preventive, or applied prescriptions. They are tied directly to the variable-importance ordering and the conditional pathways identified in the tree.

First, preoccupied attachment was the variable most strongly associated with classification into the high manipulation tendency category among adults younger than 26 years. Future longitudinal studies should examine whether elevated preoccupied attachment temporally precedes the consolidation of manipulation tendencies in this developmental window (ages 23–26), so that the directionality implied by the present classification structure can be tested rather than assumed.

Second, lower self-esteem differentiated higher-manipulation profiles within both the high- and low-preoccupied branches. Future research should therefore examine self-esteem in combination with attachment-related vulnerability rather than as an isolated correlate, ideally using designs that can model the joint contribution of these two constructs over time.

Third, the conditional gender pattern observed in a single small terminal cell (older, low-preoccupied, low-self-esteem) does not justify any general gender-based interpretation. This pattern should be revisited only in larger, gender-balanced samples that also measure gender role and cultural variables (e.g., masculinity ideology) capable of accounting for the conditional effect observed here.

Fourth, the modest internal consistency of the RSQ subscales (α = 0.60–0.66) qualifies all attachment-related findings of the present model. Replication using attachment instruments with stronger psychometric properties—such as the Experiences in Close Relationships–Revised—is necessary before the attachment-based components of the present classification structure can be considered reliable.

Fifth, the present analysis modelled the total composite score of the Manipulation Scale in Human Relations as a measure of general manipulative tendency. Future studies should test whether the same variable hierarchy applies to each of the five sub-dimensions (emotional manipulation, aggressive manipulation, victim selection, self-concealment, strategy use), which may yield distinct, subdimension-specific classification profiles.

Sixth, because single-tree classifications are known to be sensitive to small variations in training data, the specific split thresholds reported here (e.g., Preoccupied = 13; Age = 23 and 26; Self-esteem = 22) should be regarded as sample-conditional. Ensemble-based extensions—such as random forests, bagging, or boosted trees—should be applied to independent samples to assess the stability of the variable-importance ordering and the threshold values.

Finally, dyadic and cross-cultural replications would extend the present individual-level, single-sample findings. Couple-level designs that assess both partners’ attachment styles and self-esteem could clarify how the profiles identified here operate within actual relational dynamics, while cross-cultural replications could test whether the gender- and self-esteem-related patterns generalize beyond the present sociocultural context.

## 7. Limitations

Several methodological limitations should be considered when interpreting the present findings.

First, the cross-sectional and classification-based design does not permit inferences about temporal precedence, causal direction, or underlying mechanisms; the results describe associations and conditional classification patterns rather than directional effects. Relatedly, the C&RT model was developed and evaluated within a single dataset using internal train–test partitioning and 10-fold cross-validation, and has not yet been validated on an independent external sample. The reported tree structure, variable-importance ordering, and split thresholds should therefore be regarded as sample-conditional approximations rather than fixed cut-off points.

Second, several measurement-level constraints qualify the model. All variables were obtained from self-report instruments administered in a single online session, which exposes the observed associations to social-desirability bias—particularly relevant for manipulation tendency given its negative connotations—and to common-method variance arising from shared response tendencies and a common rater. Multi-source data would mitigate these concerns. Additionally, the outcome was operationalized using the total composite score of the Manipulation Scale in Human Relations rather than the emotional manipulation subdimension alone; although justified by the high internal consistency of the composite (α = 0.92) and by the study’s aim of profiling general manipulative tendency, this choice means that the present findings should be read as classification patterns associated with overall manipulative tendency, of which emotional manipulation is one component. In addition, the internal consistency of the RSQ attachment subscales was modest (α = 0.60–0.66), reflecting the brief length and heterogeneous content of the subscales. Because all four attachment dimensions functioned as discriminating variables in the model, this measurement error may have attenuated observed associations and affected the precise position of attachment-based splits; replication using instruments with stronger psychometric properties is therefore needed. The decision to dichotomize manipulation tendency via a median split, although required by the C&RT algorithm and methodologically defensible in the absence of an established clinical cut-off, represents a major limitation of the present study; it reduces statistical power, discards within-group variability, and produces an artificial high/low distinction that does not correspond to a validated diagnostic threshold. Validated clinical thresholds should be used when available.

Third, several sampling- and analysis-related constraints further qualify the findings. Participants were recruited online through convenience and snowball sampling, which likely produced a sample skewed toward digitally active and younger adults and limits representativeness. The sample was also unbalanced with respect to gender, which reduces the precision of gender-related splits, and demographic information was restricted to age and gender; relevant variables such as relationship status, socioeconomic status, education, cultural background, and history of psychological support were not assessed, which constrains contextual interpretation of the present profiles. No formal sensitivity analyses (e.g., alternative dichotomization thresholds, repeated random seeds) or external validation on an independent sample were conducted; the reported tree structure should therefore be interpreted as sample-conditional.

Fourth, the analytic approach has known methodological limits that are inherent to single decision trees. Because C&RT splits are determined greedily and locally, small variations in training data can alter the choice of splitting variables, the exact thresholds, or the order in which variables enter the tree. The conservative stopping rules, stratified partitioning, and cost-complexity pruning applied here mitigate but do not eliminate this variance. Ensemble extensions such as bagging, random forests, or boosted trees aggregate over many resampled trees and would provide a more robust assessment of the stability of the variable-importance ordering reported here. Finally, the model was restricted to the predictors examined; other contextual, relational, and personality-related variables that may be associated with manipulation tendency were not included and warrant investigation in future work.

## Figures and Tables

**Table 1:** Performance Metric.

Predicted	Actual		Metric	Formula
	Actual = 1	Actual = 0	Sensitivity (Recall)	TP/(TP + FN)
Predicted = 1	True Positive (TP)	False Positive (FP)	Specificity	TN/(TN + FP)
Predicted = 0	False Negative (FN)	True Negative (TN)	Precision	TP/(TP + FP)
			Accuracy	(TP + TN)/(TP + FP + FN + TN)
			F-score	2 × (Precision × Sensitivity)/(Precision + Sensitivity)

**Table 2:** Variable Importance Rankings in the Decision Tree Model for Manipulation Tendency Classification.

Rank	Variable	Score	Relative (%)
1	Preoccupied	68.61	32.6
2	Self-esteem	34.76	16.5
3	Age	34.33	16.3
4	Dismissive	28.48	13.6
5	Gender	21.58	10.3
6	Fearful	18.27	8.7
7	Secure	4.11	2.0

Note. Variable importance values reflect the relative contribution of predictors to the hierarchical splitting structure of the decision tree and should not be interpreted as effect sizes or indicators of causal influence.

**Table 3:** Decision Tree Performance Metrics.

Dataset	Accuracy	Sensitivity	Specificity	Precision	F1_Score
Training	0.86	0.81	0.92	0.91	0.85
Test	0.81	0.74	0.88	0.86	0.79

## Data Availability

The data are available upon request to the corresponding author.
